# Sarcoidosis and Its Splenic Wonder: A Rare Case of Isolated Splenic Sarcoidosis

**DOI:** 10.1155/2018/4628439

**Published:** 2018-11-05

**Authors:** Khushali Jhaveri, Abhay Vakil, Salim R. Surani

**Affiliations:** ^1^Department of Internal Medicine, Georgetown University Washington Hospital Center, Washington, DC, USA; ^2^University of North Texas, Denton, TX, USA; ^3^Texas A&M University, College Station, TX, USA

## Abstract

Sarcoidosis is a systemic granulomatous disease of unknown etiology characterized by presence of noncaseating granulomas in the involved organs. The pulmonary interstitium is most commonly affected but extrapulmonary involvement can occur in almost any other organ system. Such an involvement can occur with or without the presence of pulmonary involvement, but isolated extrapulmonary involvement has been noted only in around 10% of cases. Isolated splenomegaly is very rare and an uncommon presentation of sarcoidosis. It is clinically challenging because of the extensive differential diagnosis. Among the many considerations are hematologic malignancies, primary splenic or metastatic tumors, infiltrative disorders, inflammatory disorders, and infections. We hereby discuss an interesting case of a 40-year-old female with isolated splenic sarcoidosis.

## 1. Introduction

Sarcoidosis is a systemic granulomatous disease of unknown etiology characterized by presence of noncaseating granulomas in the involved organs [1]. While the pulmonary interstitium is most commonly affected [2], extrapulmonary involvement can occur in almost any other organ system [3] including the skin, eyes, and abdominal organs [2]. Such an involvement can occur with or without the presence of pulmonary involvement [4]. Isolated extrathoracic sarcoidosis is reported in only 10% of cases [5]. Splenic involvement has been reported in about 40% cases of multisystem sarcoidosis [6], but isolated splenic involvement in sarcoidosis without any radiographic or clinical pulmonary involvement is extremely rare [7]. To the best of our knowledge, only 10 such cases of isolated splenic sarcoidosis have been reported in the current literature. Here, we present a case of a 40-year-old female presenting with constitutional symptoms found to have isolated splenomegaly with multiple hypodense splenic lesions and no other multisystem involvement.

## 2. Case Report

A healthy, 40-year-old female born in the Dominican Republic living in the United States for past 20 years presented with a complaint of low-grade fevers with temperature max. of 100.2°F, night sweats, malaise, and fatigue for 6-week duration. The patient denied having any rash, exertional dyspnea, cough, or joint pains. She denied any recent travel, sick contacts, or recent changes in weight and appetite. She did not recall any significant occupational, chemical, or animal exposure. The patient also denied having any risk factors for HIV.

**Figure 1:**
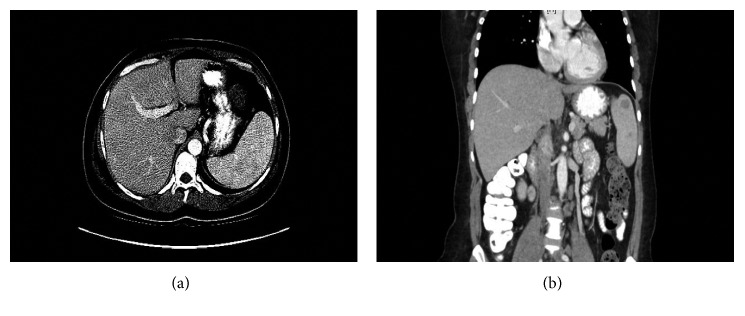
(a, b) CT scan of the abdomen showing presence of an enlarged spleen measuring 16 × 7 × 6 cm with multiple hypodense lesions.

On physical examination, the patient was afebrile with normal vital signs. Abdominal examination revealed a palpable spleen without other appreciable organomegaly. There was no tenderness, guarding, or rigidity. Her chest, cardiovascular, genital, neurologic, and extremities examinations were unremarkable. Initial laboratory studies revealed a leukocyte count of 15,000 × 10^9^/L with an absolute eosinophil count of 800 cells × 10^9^/L (normal <500 × 10^9^/L). All the other parameters of the complete blood count and differential markers of renal and hepatic function including serum calcium were within normal limits. A chest radiograph failed to demonstrate any consolidation, effusion, or cardiomegaly. Abdominal ultrasound was done and was nondiagnostic. Abdominal computed tomography (CT) imaging revealed the presence of an enlarged spleen measuring 16 × 7 × 6 cm with multiple hypodense lesions (Figures 1(a) and 1(b)). The liver was reported to be normal without any evidence of hepatomegaly. No lymphadenopathy was seen on the imaging studies.

The patient's systemic symptoms with splenomegaly with multiple hypodense splenic lesions raised high suspicion for a primary hematologic malignancy or a primary splenic tumor. Following a normal peripheral blood smear and a normal bone marrow examination, a full-body positron emission tomography (PET) scan was performed. PET scan showed multiple hypermetabolic splenic lesions with an SUV of 13.0 and no pathologic uptake in any other organ or lymph nodes.

**Figure 2:**
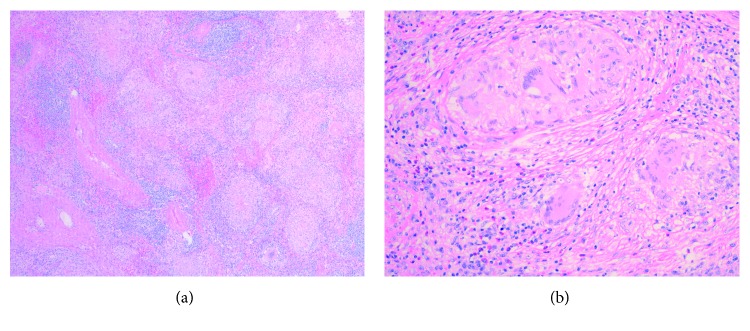
(a, b) Low- and high-power slides showing multiple noncaseating granulomas with multiple histiocyte-consisting follicles and absence of central necrosis or evidence of polynuclear neutrophils.

To establish a specific diagnosis, the patient underwent laparoscopic splenectomy. Histopathologic examination of the resected tissue showed multiple noncaseating granulomas with multiple histiocyte-consisting follicles (Figures 2(a) and 2(b)). There was no central necrosis or evidence of polynuclear neutrophils. Special staining for acid-fast bacilli and fungus was negative. GMS was done and was negative. Angiotensin-converting enzyme (ACE) levels were checked and were found to be 75 units/L (normal 8–53 units/L). Detailed history failed to reveal any environmental or occupational exposure to beryllium and talc.

The patient failed to show any evidence of thoracic and other organ system involvement at the time of diagnosis. She did well after splenectomy with subsequent resolution of her systemic symptoms and normalization of her serum ACE levels over the next 3 months. She presented with erythema nodosum-like skin lesions after 6 months of her splenectomy. The skin lesions resolved spontaneously. She did not require any medical treatment for sarcoidosis. She continues to be asymptomatic without any evidence of thoracic and other organ system involvement 2 years after splenectomy.

## 3. Discussion

Isolated splenomegaly with systemic symptoms is unusual and clinically challenging. Differential diagnoses can include hematologic malignancies, primary splenic or metastatic tumors, infiltrative splenic disorders like amyloidosis and Langerhans cell histiocytosis, inflammatory disorders like sarcoidosis, systemic lupus erythematous, and rheumatoid arthritis, and infections like tuberculosis, fungi, and parasites. Extrathoracic involvement, in particular in the liver and spleen, can be isolated without constitutional symptoms or systemic disease [8]. Isolated splenic sarcoidosis is usually asymptomatic [6], though when present, abdominal pain, fever, malaise, and weight loss are the most common symptoms in the patients with sarcoidosis of spleen [9, 10]. Our patient presented with a complaint of low-grade fevers, night sweats, malaise, and fatigue for 6 weeks. Absence of multisystem involvement made amyloidosis and Langerhans cell histiocytosis unlikely. Splenomegaly in rheumatoid arthritis is often associated with neutropenia which was absent in our patient. Similarly, the absence of lymphadenopathy, negative bacterial and fungal blood cultures, negative AFB and GMS, negative interferon testing for tuberculosis and a negative travel history to the endemic areas for malaria and leishmaniasis made each of these acute infections highly unlikely.

Laboratory studies are usually nondiagnostic but might be characterized by anemia, thrombocytopenia, or neutropenia. A linear relationship has been reported between the serum ACE level and spleen size. However, cases with a normal ACE level have been reported. In our patient, angiotensin-converting enzyme (ACE) levels were checked and were found to be 75 units/L (normal 8–53 units/L).

Extrapulmonary sarcoidosis can show nonspecific findings on imaging. CT, MRI, PET-CT, and CEUS [8] have a great potential in the assessment of focal lesions in sarcoidosis. Hypoechoic and hyperechoic lesions but also isoechoic masses can be clearly highlighted after injecting the contrast agent [8]. In our patient, systemic symptoms with splenomegaly with multiple hypodense splenic lesions raised high suspicion for a primary hematologic malignancy or a primary splenic tumor. Following a normal peripheral blood smear and a normal bone marrow examination, PET scan was performed. PET scan showed multiple hypermetabolic splenic lesions and no pathologic uptake in any other organ or lymph nodes. Although splenic size and splenic lesions can be characterized by imaging, histopathologic examination is required for definitive diagnosis. PET-CT does not help in differentiation of malignant lesions from sarcoidosis [11].

To establish a specific diagnosis, we decided to proceed with laparoscopic splenectomy which on histopathologic examination of the resected tissue showed multiple noncaseating granulomas with multiple histiocyte-consisting follicles (Figures 2(a) and 2(b)). There was no central necrosis or evidence of polynuclear neutrophils. Special staining for acid-fast bacilli and fungus was negative.

Patients with splenic sarcoidosis should also be evaluated for pulmonary and other organ system involvement at the time of diagnosis and subsequent follow-up. Immunosuppressive drugs like steroids, methotrexate, and azathioprine should be considered in patients with isolated splenic sarcoidosis when systemic symptoms are persistent. About 60% of all symptomatic patients are known to show spontaneous remission [9]. Asymptomatic patients do not require treatment and should be followed closely. Asymptomatic patients with isolated splenic involvement usually have a good prognosis without any medical therapy [12].

Splenectomy should be considered in the patients with symptomatic splenomegaly not responding to immunosuppressive therapy, severe hypersplenism, as a prophylaxis for splenic rupture and for histopathologic examination in the cases where the diagnosis is uncertain, and there is a high suspicion of malignant involvement of the spleen [13]. Splenectomy does not change the natural history of sarcoidosis, and patients can develop pulmonary and/or systemic sarcoidosis even after removal of the spleen.

## 4. Conclusion

Sarcoidosis of the spleen, although rare, should be considered in the differential diagnosis of the patients presenting with systemic symptoms and splenomegaly. Histologic examination of the spleen is necessary for establishing the diagnosis of splenic sarcoidosis. All the patients with an established diagnosis of isolated splenic sarcoidosis should be evaluated for thoracic and other organ system involvement at the time of diagnosis and at subsequent follow-up. Immunosuppressive therapy should be reserved only for symptomatic patients with splenic sarcoidosis who fails to resolve spontaneously.
